# Acute styrene exposure induces hepatocellular injury and molecular stress responses in *Oryzias celebensis*: Evidence for a tropical sentinel species in ecotoxicological monitoring

**DOI:** 10.14202/vetworld.2025.3902-3913

**Published:** 2025-12-14

**Authors:** Amelia Ramadhani Anshar, Huda Shalahudin Darusman, Wasmen Manalu, Khusnul Yaqin, Muhammad Ardiansyah Nurdin, Muhammad Reza Cordova

**Affiliations:** 1 Animal Biomedical Sciences Study Program, School of Veterinary and Biomedical Sciences, IPB University, Bogor, 16680, Indonesia; 2 Veterinary Medicine Study Program, Hasanuddin University, Makassar, 90245, Indonesia; 3 Division of Pharmacology and Toxicology, School of Veterinary and Biomedical Sciences, IPB University, Bogor, 16680, Indonesia; 4 Primate Research Center, Institute of Research and Community Service, IPB University, Bogor, 16151, Indonesia; 5 Division of Physiology, School of Veterinary and Biomedical Sciences, IPB University, Bogor, 16680, Indonesia; 6 Aquatic Resources Management Study Program, Hasanuddin University, Makassar, 90245, Indonesia; 7 Research Center for Oceanography, National Research and Innovation Agency, Jakarta, 14430, Indonesia; 8 Research Center for Oceanology, National Research and Innovation Agency, Tangerang Selatan, 15314, Indonesia

**Keywords:** CYP1A1, ecotoxicology, hepatotoxicity, inflammation, molecular biomarkers, *Oryzias celebensis*, oxidative stress, plastic-derived pollutants, styrene toxicity, tropical freshwater fish

## Abstract

**Background and Aim:**

Plastic-derived monomers such as styrene are increasingly detected in tropical freshwater ecosystems at concentrations approaching 0.8 mg/L. These contaminants pose toxicological risks to aquatic organisms, particularly through oxidative stress, inflammation, and metabolic disruption. *Oryzias celebensis* (Celebes medaka), an endemic tropical freshwater species, offers high ecological relevance for assessing pollutant impacts in Southeast Asia. This study evaluated the hepatic histopathological and molecular responses of *O. celebensis* following acute styrene exposure and assessed its suitability as a tropical sentinel species.

**Materials and Methods:**

Adult *O. celebensis* were exposed to 0.1, 0.25, 0.5, and 0.75 mg/L styrene for 96 h under semi-static conditions. Liver tissues were examined for cytoplasmic vacuolization, necrosis, inflammatory cell infiltration, and congestion using standard histopathological scoring. Expression of biomarker genes related to detoxification (cytochrome P450 1A1 [CYP1A1]), oxidative stress (catalase, superoxide dismutase [SOD]), and inflammation (tumor necrosis factor [TNF]) was quantified using reverse transcription quantitative polymerase chain reaction. Correlations between biomarker expression and tissue lesions were analyzed using Spearman’s coefficients.

**Results:**

Styrene exposure induced concentration-dependent hepatic injury. Histopathological lesions intensified markedly at ≥0.5 mg/L, with prominent vacuolization, necrosis, and inflammatory infiltration. CYP1A1 was strongly upregulated, showing a 36.9-fold increase at 0.75 mg/L (p < 0.01), indicating robust activation of the aryl hydrocarbon receptor (AhR) pathway. Antioxidant enzymes (catalase and SOD) and TNF expression also increased significantly, reflecting oxidative and inflammatory stress. Strong positive correlations (r_s_ = 0.93–0.99) were observed between gene expression and lesion severity, confirming mechanistic links between molecular responses and tissue pathology.

**Conclusion:**

Acute styrene exposure triggers coordinated hepatocellular injury and molecular stress responses in *O. celebensis* through activation of aryl hydrocarbon receptor-CYP1A1, nuclear factor erythroid 2-related factor 2, and nuclear factor kappa-light-chain-enhancer of activated B cells pathways. The strong correspondence between histopathological and transcriptional biomarkers demonstrates that this species is highly sensitive to styrene toxicity and suitable for ecotoxicological monitoring. Findings highlight the need for environmental surveillance of industrial pollutants in tropical freshwater systems.

## INTRODUCTION

The accumulation of plastic waste in aquatic ecosystems has become an increasingly urgent global environmental concern. The massive production and short-term use of plastic materials have led to the buildup of plastic debris, which gradually degrades into microplastics and releases toxic chemical constituents, including styrene monomers, over time [1, 2]. Styrene, a volatile organic compound extensively used in the manufacturing of polystyrene products, is known to leach into the environment from landfills, industrial waste, and plastics exposed to ultraviolet radiation [3]. These pollutants pose significant risks to aquatic organisms due to their bioaccumulative and toxic properties [4].

Recent field investigations in Southeast Asia have reported styrene concentrations ranging from 0.03 mg/L to 0.8 mg/L in rivers and estuaries near polymer-manufacturing and waste-processing sites [5, 6]. These values often exceed the recommended limits set by the World Health Organization (WHO, <0.02 mg/L) and the United States Environmental Protection Agency (USEPA) for drinking water (<0.1 mg/L) [7, 8]. In freshwater fish, laboratory findings indicate that even sub-milligram levels of styrene can disrupt endocrine functions, impair growth, and induce hepatic injury [4]. Styrene can enter aquatic organisms through dermal absorption, ingestion, or trophic transfer, triggering oxidative stress, DNA damage, and inflammatory responses mediated by the aryl hydrocarbon receptor (AhR)-CYP1A1 and nuclear factor erythroid 2-related factor 2 (Nrf2)-nuclear factor kappa-light-chain-enhancer of activated B cells (NF-κB) pathways [5, 6]. Styrene’s small molecular size facilitates tissue penetration, leading to systemic and immunological effects [7, 8], while co-exposure with plastic particles amplifies reactive oxygen species (ROS) generation by up to 40% compared with styrene alone [4]. Despite its recognized toxicity, most mechanistic studies remain restricted to temperate species, leaving a substantial data gap in tropical ecosystems where degradation kinetics and biological sensitivity differ significantly. To address this knowledge gap, the present study integrates histopathological and molecular biomarker approaches to elucidate styrene-induced hepatotoxicity in *Oryzias celebensis*, a tropical freshwater species endemic to Sulawesi, thereby establishing it as a novel sentinel organism for ecotoxicological assessment and contributing essential data for ecological risk evaluation in tropical freshwater environments.

Effective assessment of systemic toxicity induced by styrene requires a sensitive, ecologically relevant model organism. *O. celebensis* (Celebes medaka), an endemic species to Sulawesi freshwater habitats, offers such suitability. Unlike the temperate *O. latipes* or estuarine *Oryzias javanicus*, *O. celebensis* is a true tropical freshwater species that naturally inhabit Sulawesi’s inland rivers and lakes. Its physiological adaptation to warm, low-salinity environments makes it an ecologically relevant indicator of tropical water systems, where temperature and pollutant degradation rates differ substantially from those in temperate regions. This species is characterized by a small body size, short life cycle, and ease of biological observation, making it a valuable model for ecotoxicological studies [9]. Notably, its sensitivity to various pollutants further validates its potential as a bioindicator [6, 7, 10]. Despite these advantages, *O. celebensis* has not been studied in detailed mechanistic studies of the toxicity of plastic-derived monomers. Conversely, its congener *Oryzias latipes* has been extensively used to elucidate oxidative stress and inflammatory signaling pathways [6, 10].

Although styrene is a well-documented pollutant associated with plastic degradation, most existing toxicological research has focused on temperate model species such as *O. latipes* and *Danio rerio*, leaving a critical gap in understanding its mechanistic effects on tropical freshwater organisms. Current evidence on styrene toxicity in tropical ecosystems is largely limited to environmental monitoring data, while comprehensive studies integrating histopathology, oxidative stress markers, detoxification pathways, and inflammatory responses are scarce. Moreover, tropical freshwater environments differ markedly from temperate systems in temperature, microbial activity, and degradation kinetics, all of which may alter pollutant bioavailability and organismal sensitivity. Despite the ecological relevance of *O. celebensis*, a tropical endemic species with demonstrated physiological sensitivity, its potential as a sentinel organism for plastic-derived pollutants remains largely unexplored. No previous research has examined the coordinated activation of the AhR–CYP1A1, Nrf2, and NF-κB pathways or their relationship with hepatic structural damage in this species. Therefore, there is a significant knowledge gap regarding how acute styrene exposure affects tropical fish at both molecular and tissue levels, and how these mechanistic endpoints can inform ecological risk assessments in tropical freshwater systems.

To address the identified gap, the present study aimed to evaluate the hepatotoxic and molecular responses of *O. celebensis* following acute exposure to environmentally relevant concentrations of styrene. Specifically, the study sought to (i) characterize concentration-dependent histopathological alterations in the liver, including vacuolization, necrosis, congestion, and inflammatory infiltration; (ii) quantify transcriptional changes in key biomarker genes associated with detoxification (CYP1A1), oxidative stress (catalase and superoxide dismutase [SOD]), and inflammation (tumor necrosis factor [TNF]) using reverse transcription quantitative polymerase chain reaction (RT-qPCR); and (iii) determine the relationship between molecular biomarker activation and tissue lesion severity to elucidate mechanistic pathways underlying styrene-induced hepatotoxicity. By integrating structural and molecular endpoints, this study aimed to validate *O. celebensis* as a sensitive tropical sentinel species and provide mechanistic evidence for its use in ecotoxicological monitoring and environmental risk assessment of plastic-derived contaminants in Southeast Asian freshwater ecosystems.

## MATERIALS AND METHODS

### Ethical approval

All experimental procedures involving *O. celebensis* were reviewed and approved by the Health Research Ethics Committee, Faculty of Medicine, Hasanuddin University, under Protocol No. UH24060405. The study was conducted in full compliance with the institutional guidelines for the care and use of research animals and adhered to the Animal Research: Reporting of *In Vivo* Experiments (ARRIVE) 2.0 standards. All handling, acclimation, exposure, sampling, and euthanasia procedures followed the ethical principles established by the Organization for Economic Co-operation and Development (OECD) Test Guideline 203 for fish acute toxicity testing.

To ensure humane treatment, wild adult *O. celebensis* were collected under local authority permission, transported under minimal-stress conditions, and acclimatized for 4 weeks before experimentation. Only healthy individuals free of lesions or parasites were included. Throughout the study, water quality, photoperiod, and environmental parameters were rigorously monitored to minimize stress. During the 96-h exposure, fish were observed regularly for behavioral abnormalities, and any individuals exhibiting signs of severe distress were humanely euthanized.

At the end of the experimental period, all fish were euthanized using β-hydroxyethylphenyl ether (MS-222 alternative) at a concentration that resulted in rapid loss of equilibrium and cessation of opercular activity, in accordance with international humane endpoints for small fish models. No unnecessary harm, invasive procedures, or stressful manipulations were performed. All efforts were made to reduce animal use by employing statistically justified sample sizes and maximizing biological information obtained per specimen.

The experimental facility is certified for aquatic toxicology research and operates under biosafety and animal welfare oversight of the Veterinary Medicine Study Program, Faculty of Medicine, Hasanuddin University. No endangered or protected species were used, and no live fish were released back into natural habitats following exposure.

### Study period and location

The study was conducted from March 2024 to January 2025. Wild adult *O. celebensis* were collected from the Salarang River, Maros, South Sulawesi, Indonesia, and subsequently acclimated in the laboratory. All styrene exposure experiments, necropsy procedures, and histopathological processing were performed at Laboratory of Integrated, Veterinary Medicine, Universitas Hasanuddin. Water quality measurements were conducted at Laboratory of Aquatic Resource Management, Universitas Hasanuddin. Molecular analyses were carried out at the Biotechnology Laboratory, Primate Research Center, IPB University.

### Experimental design and test organisms

Wild adult *O. celebensis* were collected from a freshwater habitat at the Salarang River in Maros, South Sulawesi, and subsequently acclimatized for 4 weeks under controlled laboratory conditions to ensure homogeneity in age and size. A total of 75 fish (15/treatment group) were maintained in 35-L aquaria under controlled conditions (temperature: 26°C ± 1°C; pH: 7.4; dissolved oxygen: 7.2 ± 0.2 parts per million; photoperiod: 14:10 h light–dark cycle). Fish were acclimatized for 4 weeks and fed a combination of Otohime B1 and artemia (5% body weight, three times daily). All fish were externally inspected for visible parasites or lesions, and only healthy individuals were included. Acclimation mortality was <5%, indicating successful adaptation to laboratory conditions.

### Styrene and solvent preparation

Analytical-grade styrene monomer (≥99% purity; Merck, Germany) was used in all treatments. The stock solution was prepared fresh daily by dissolving styrene in absolute ethanol (0.5% v/v) and stored in amber glass bottles at 4°C to minimize volatilization and photodegradation. Working concentrations (0.1, 0.25, 0.5, and 0.75 mg/L) were prepared by dilution with dechlorinated water immediately before exposure. All solutions were thoroughly mixed for 5 min before use to ensure homogeneity. All experimental tanks were made of borosilicate glass and tightly covered during exposure to prevent adsorption or loss of styrene. The ethanol level (0.5% v/v) was selected based on pre-exposure range-finding tests showing no behavioral, histopathological, or transcriptional changes compared with the control group. A solvent control (0.5% ethanol without styrene) was included in parallel and was biologically inert.

### Styrene exposure treatments

Fish were randomly assigned into five groups (3 tanks per treatment, 5 fish per tank; total n = 15): one control group (0.5% ethanol) and four treatment groups receiving styrene concentrations of 0.1, 0.25, 0.5, and 0.75 mg/L. Fish were randomly assigned to tanks, and tank positions were randomized within the experimental area to minimize environmental variability. The tank was used as the experimental unit for statistical analyses. All histopathological examinations and gene expression analyses were performed under blinded conditions, with independent analysis of coded samples to avoid observer bias.

A preliminary range-finding test was conducted to determine the 96-h lethal concentration 50% (LC_50_) value for styrene in *O. celebensis*. The fish were exposed to concentrations ranging from 0.05 mg/L to 2.0 mg/L (n = 15/group; triplicate tanks) under semi-static conditions. Mortality and behavioral responses were recorded at 24-h intervals. Based on probit analysis, the 96-h LC_50_ was estimated at 0.92 mg/L, and sublethal concentrations (0.1–0.75 mg/L) were selected for the present study.

Each group was maintained in individual 15 × 15 × 20 cm aquaria for a 96-h exposure period without feeding. The fish were fasted during the 96-h exposure period to prevent water contamination and ensure uniform metabolic activity across treatments. Behavioral and physiological observations (swimming activity, balance, opercular rate, and surface behavior) were recorded at 6-h intervals.

During the exposure period, semi-static renewal methods ensured consistent styrene concentrations, which were validated daily by gas chromatography-mass spectrometry analysis confirming <10% variation between initial and post-renewal measurements. Water parameters (temperature, pH, dissolved oxygen, ammonia, nitrite, and nitrate) were monitored daily using calibrated portable meters and colorimetric test kits. Ammonia, nitrite, and nitrate levels were maintained below 0.05, 0.02, and 0.2 mg/L, respectively. The test media were maintained with 80% water renewal every 24 h to ensure chemical stability and water quality. All aquaria were covered with glass lids to minimize volatilization, and aeration was provided through narrow tubing to limit headspace exposure. Temperature, pH, and dissolved oxygen were monitored daily to ensure stable environmental conditions throughout the experiment.

### Histopathological analysis

Fish were anesthetized with β-hydroxyethylphenyl ether (200 mg/L) until loss of equilibrium and opercular movement were observed, then dissected. Liver tissues (3 fish per treatment) were fixed in 10% buffered neutral formalin for 48 h, processed through standard histological techniques (paraffin embedding, sectioning at 5–6 µm, and hematoxylin and eosin staining), and examined under a light microscope (Olympus BX50F4, Olympus Optical Co. LTP, Japan) at magnifications of 100× and 400×. Tissue damage was scored semi-quantitatively for vacuolization, necrosis, inflammatory infiltration, and congestion based on standard scoring criteria [11, 12]. Lesions were semi-quantitatively scored on a five-point scale: 0 = Absent (≤5% affected area), 1 = Minimal (5%–25%), 2 = Mild (26%–50%), 3 = Moderate (51%–75%), and 4 = Severe (>75%).

### Gene expression analysis

Biological replicates consisted of five fish per tank (3 tanks per treatment). Liver tissues were individually extracted and then pooled per tank (n = 3 pools per treatment) for RT-qPCR. Each pool was assayed in triplicate. For gene expression, total RNA (1.9–2.1 µg/µL) was isolated, and complementary DNA (cDNA) was synthesized in 20 µL reactions. Liver tissue from each fish group was pooled for RNA extraction using the Quick-RNA MiniPrep Kit (Zymo Research, USA). RNA purity and concentration were measured using a NanoDrop spectrophotometer (A260/280 = 1.9–2.1) and RIN >7. cDNA was synthesized using the ReverTra Ace quantitative polymerase chain reaction (qPCR) Kit (Toyobo, Japan). Primer validation included melting curve analysis and amplification efficiency (90%–110%). The synthesized cDNA was stored at −20°C until further analysis.

Each gene expression assay was performed in technical triplicate, in accordance with MIQE guidelines. RT-qPCR using SensiFAST SYBR No-ROX Kit (Bioline, UK) was conducted on a CFX Opus 96 Real-Time PCR System (Bio-Rad, USA). Each 20 µL reaction contained 10 µL SYBR Master Mix, 0.4 µL of each primer (10 µM), 2 µL of cDNA, and nuclease-free water. The thermal profile consisted of 95°C for 2 min, followed by 40 cycles of 95°C for 5 s, 60°C for 10 s, and 72°C for 20 s. Melt-curve analysis (65°C–95°C) verified amplification specificity. Relative gene expression (CYP1A1, catalase, SOD, and TNF) was calculated using the 2^–ΔΔCt^ method [12, 13]. β-actin served as the internal control after confirming its stability across groups. Primers were designed using Primer3 software based on National Center for Biotechnology Information GenBank sequences, as shown in Table 1 [14–17].

**Table 1:** Primer sequences used for RT-PCR analysis. Based on available sequences in GenBank, the selected genes are associated with detoxification activity (CYP1A1), oxidative stress (catalase and SOD), and immune response (TNF).

Gene target	Primer pair (5’–3’)	Annealing temperature (°C)	GenBank (RefSeq)	Product size
Beta-actin	F: TACCGCTGCCTCTTCTTCAT R: ACAGGTCCTTACGGATGTCG	53 53	FJ754999	693
Cyp1A1	F: CGACTTTTCCGGCAGGC R: GCCAACTTTCTGCGGGC	57 57	EF535032	516
Catalase	F: TGTTTGAACACGTTGGGAAA R: ATGGAAAAAGCATTGCATCC	53 53	XM_015388787	2554
SOD3	F: CTGCTCTGCTGCTGTTTTTG R: GCTGCATACAGGGTTCCATT	54 54	XM_015394947	1455
TNF	F: GTGGGAAATGAGGATCAGGA R: GCTTGAAAGCACCCAAGAAG	52 52	XM_015393156	2822

β-actin was used as the internal reference gene [14–17]. RT-PCR = Reverse transcription-polymerase chain reaction, SOD = Superoxide dismutase, TNF = Tumor necrosis factor, F = Forward, R = Reverse.

### Statistical analysis

The tank was considered the experimental unit for histopathological analysis (n = 3 per treatment), whereas each pooled liver sample (from three fish) represented one biological replicate for qPCR (n = 3 per treatment). Data were analyzed using GraphPad Prism version 10.1 (GraphPad Software, San Diego, CA, USA). Kruskal–Wallis tests were used for group comparisons, followed by Dunn’s multiple comparison test. Spearman’s rank correlation coefficients (r) were calculated toevaluate relationships between gene expression and histopathological scores.

## RESULTS

### Histopathological alterations

#### Overall liver damage pattern

**Figure 1:**
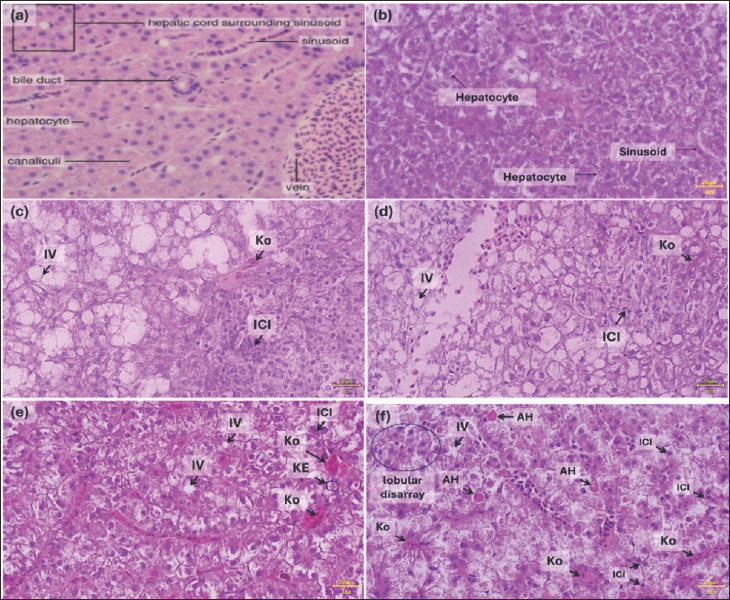
Histological appearance of liver tissue in *Oryzias celebensis* (Celebes medaka) following exposure to various concentrations of styrene. (a) Normal liver morphology in fish [9]. (b) Control group (0.5% ethanol) showing hepatocytes with clearly defined nuclei and homogeneous cytoplasm. (c) Styrene (0.1 mg/L) and styrene (0.25 mg/L): initial cytoplasmic vacuolization (IV), neutrophil infiltration (ICI), and mild congestion (Ko) are observed. (e) 0.5 mg/L styrene and (f) 0.75 mg/L styrene: more extensive IV, pronounced Ko, karyorrhexis, apoptotic hepatocytes, and widespread ICI are visible, accompanied by loss of normal sinusoidal architecture (circled areas). Hematoxylin and eosin staining; magnification 40×; scale bar = 20 µm.

Histological analysis revealed that 96-h styrene exposure induced acute, progressive liver damage, with increasing severity corresponding to higher concentrations (Table 2). In the control group, hepatic lobular architecture appeared normal, with orderly hepatocytes, centrally located round nuclei, and central veins. In contrast, representative photomicrographs (Figure 1) [9] show progressive hepatic alterations with increasing styrene concentration. The 0.5 mg/L and 0.75 mg/L styrene-treated groups exhibited marked cytoplasmic vacuolization, focal necrosis, hepatocyte cell damage, and extensive inflammatory cell infiltration (Figure 1) [9]. These changes were more pronounced at 0.5 mg/L and 0.75 mg/L of styrene exposure, consistent with concentration-dependent hepatocellular damage.

**Table 2:** Semi-quantitative liver lesion scores (mean ± SEM) after 96 h of styrene exposure in *Oryzias celebensis* (n = 3 fishes per treatment).

Concentration (mg/L)	Vacuolization	Necrosis	Inflammatory infiltration	Cell congestion
Control	0.01 ± 0.1	0 ± 0	0.10 ± 0.01	0 ± 0
0.1	1.67 ± 0.5	1 ± 0.1	1 ± 0.11	0.66 ± 0.57
0.25	1.66 ± 0.55	3.34 ± 0.57	2.66 ± 0.57	1.33 ± 0.57
0.5	1.66 ± 0.57	3.33 ± 0.57	3.33 ± 0.57	2.66 ± 0.58
0.75	3.34 ± 0.57	4 ± 0.01	4 ± 0.01	3.34 ± 0.5

SEM = Standard error of the mean.

### Congestion and cellular degeneration

Semi-quantitative scoring indicated early signs of congestion at 0.1 mg/L, which became significant at 0.5 mg/L and 0.75 mg/L. This congestion likely resulted from microcirculatory disturbances caused by damage to peroxidative endothelial cells. Cytoplasmic vacuolization suggests that organelle dysfunction, particularly of mitochondria and the endoplasmic reticulum (ER), may be linked to disrupted lipid metabolism.

#### Inflammation and necrosis

Significant inflammatory cell infiltration observed at ≥0.25 mg/L indicates an immunological response to hepatocellular injury. This may involve activation of the NF-κB signaling pathway, which promotes the release of pro-inflammatory cytokines, such as TNF-alpha. Necrotic lesions, appearing from 0.25 mg/L, intensified at higher concentrations, indicative of severe oxidative stress and compromised membrane integrity.

### Molecular response

#### Activation of detoxification pathways (CYP1A1)

**Figure 2:**
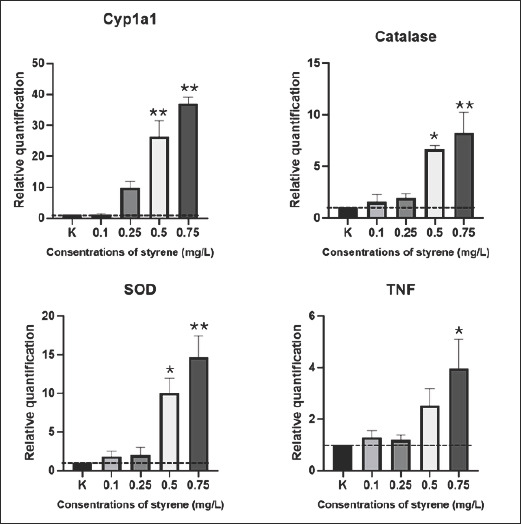
Relative transcriptional expression of the cytochrome P450 1A1, catalase, superoxide dismutase, and tumor necrosis factor genes in Celebes medaka (*Oryzias celebensis*) following a 96 h exposure to graded concentrations of styrene. Data were normalized to the unexposed control (mean ± standard error of the mean, n = 5). The dashed horizontal line denotes the baseline expression level in the control group. *p < 0.05 and **p < 0.01 indicate statistically significant and highly significant differences, respectively.

RT-qPCR results (Figure 2) demonstrated a clear, concentration-dependent upregulation of target genes. CYP1A1 expression showed an expression level of 1.00 ± 0.00 in the control group and slightly increased to 1.04 ± 0.61 at 0.1 mg/L. A sharp elevation was observed at higher concentrations, with levels reaching 9.73 ± 3.54 at 0.25 mg/L and 26.29 ± 9.04 at 0.5 mg/L. The highest induction (up to 36.9-fold at 0.75 mg/L, p < 0.01), signifying strong activation of phase I detoxification through the AhR signaling pathway, was observed.

#### Oxidative stress response (catalase and SOD)

Catalase and SOD, which are key antioxidants, were also significantly elevated, reflecting cellular efforts to counteract styrene-induced ROS production. In line with the activation of detoxification pathways, the expression levels of two key antioxidant enzymes, catalase and SOD, were also significantly upregulated. Catalase expression (Table 3) increased from 1.54 ± 1.25 and 1.93 ± 0.70 at low exposure levels (0.1–0.25 mg/L), followed by a more pronounced elevation at 0.5 mg/L (6.64 ± 0.69) and 0.75 mg/L (8.26 ± 3.41). Similarly, SOD expression exhibited a progressive response, rising from 1.84 ± 1.25 and 2.00 ± 1.72 at initial exposures to 10.02 ± 3.39 and 14.68 ± 4.68 at 0.5 and 0.75 mg/L, respectively.

**Table 3:** Relative fold-change (mean ± SEM, 95% CI) and significance levels of hepatic gene expression in *Oryzias celebensis* after 96 h of styrene exposure (n = 3 replicates).

Gene	Control	0.1 mg/L	0.25 mg/L	0.5 mg/L	0.75 mg/L	95% CI
CYP1A1	1.00	1.04 ± 0.61	9.73 ± 3.54	26.29 ± 9.04**	36.9 ± 3.87**	−29.03–12.4
Catalase	1.00	1.54 ± 1.25	1.93 ± 0.70	6.64 ± 0.69*	8.26 ± 3.41**	−9.2–3.9
SOD	1.00	1.84 ± 1.25	2.00 ± 1.72	10.02 ± 3.39*	14.68 ± 4.68**	−21.08–6.4
TNF	1.00	1.29 ± 0.46	1.19 ± 0.33	2.53 ± 1.11	3.96 ± 1.98*	−5.1–2.9

Data are presented as mean ± SEM. Fold-change values were normalized to β-actin and control group expression levels. Statistical analysis was conducted using the Kruskal–Wallis test followed by Dunn’s *post hoc* comparison. An asterisk (*) indicates a significant difference (p < 0.05), and a double asterisk (**) indicates a highly significant difference (p < 0.01). SEM = Standard error of mean, SOD = Superoxide dismutase, TNF = Tumor necrosis factor, CI = Confidence interval.

#### Inflammatory activation (TNF)

In addition to detoxification and antioxidant pathways, the immune response was activated, as evidenced by the upregulation of the pro-inflammatory cytokine gene TNF. Mean TNF expression levels increased gradually from 1.29 ± 0.46 and 1.19 ± 0.33 at lower exposures, rising further to 2.53 ± 1.11 at 0.5 mg/L and reaching 3.96 ± 1.98 at 0.75 mg/L.

### Correlation analysis

**Figure 3:**
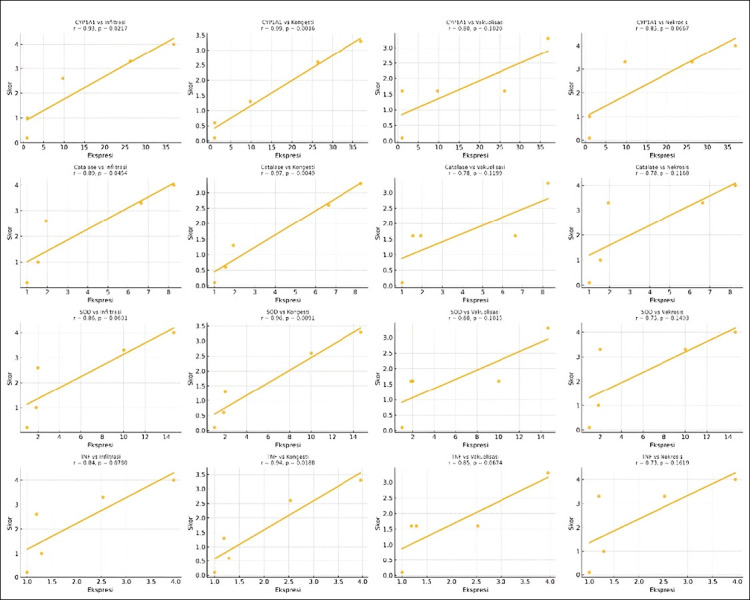
Correlation scatter plots between biomarker gene expression and histopathological scores for inflammatory cell infiltration, congestion, vacuolization, and necrosis in the liver of Celebes medaka (*Oryzias celebensis*).

Pearson r_s_ in Table 4 and scatter plot (Figure 3) confirmed strong positive relationships between gene expression levels and histological damage scores. CYP1A1 expression was most strongly correlated with liver congestion (r = 0.99, p = 0.0016), supporting its central role in initiating metabolic responses to xenobiotic stress. TNF expression was highly correlated with necrosis scores (r = 0.99, p = 0.0011), indicating that inflammation is a key contributor to cell death. These findings elucidate the role of the interconnection of molecular pathways and tissue-level outcomes in styrene hepatotoxicity.

**Table 4:** Spearman’s correlation coefficients (rₛ) between gene expression and histopathological scores.

Type of damage	CYP1A1	Catalase	SOD	TNF
Inflammatory cell infiltration	0.93 (p = 0.0217)	0.89 (p = 0.0454)	0.86 (p = 0.0601)	0.96 (p = 0.0780)
Interpretation	Very strong, significant	Very strong, significant	Very strong, significant	Very strong, significant
Congestion	0.99 (p = 0.0016)	0.97 (p = 0.0049)	0.96 (p = 0.0091)	0.97 (p = 0.0188)
Interpretation	Very strong, highest significance	Very strong, highest significance	Very strong, highest significance	Very strong, highest significance
Vacuolization	0.80 (p = 0.1020)	0.78 (p = 0.1199)	0.80 (p = 0.1015)	0.95 (p = 0.0674)
Interpretation	Strong, significant	Strong, significant	Strong, significant	Strong, significant
Necrosis	0.78 (p = 0.0667)	0.78 (p = 0.1168)	0.75 (p = 0.1403)	0.99 (p = 0.1619)
Interpretation	Strong, significant	Strong, significant	Strong, significant	Strong, significant

Exact p-values are provided to indicate the strength and significance of the associations. SOD = Superoxide dismutase, TNF = Tumor necrosis factor.

## DISCUSSION

### Overall toxicological impact of styrene

This study reinforces the toxicological impact of styrene on hepatic integrity, demonstrating that even short-term exposure can lead to notable histopathological and molecular alterations. These findings align with established evidence that styrene and its oxidative metabolites, particularly styrene-7,8-oxide (SO), disrupt hepatic microcirculation and cellular homeostasis through oxidative stress and inflammatory pathways. SO induces mitochondrial dysfunction, lipid peroxidation, and apoptosis in hepatic cells, thereby significantly contributing to liver damage [18–20]. These effects contribute to impaired endothelial integrity and vascular permeability, thereby promoting hepatic congestion and microvascular collapse. Vascular changes are considered early markers of hepatotoxic response, preceding visible parenchymal damage. Oxidative damage targets key organelles, including mitochondria and the ER, resulting in impaired lipid metabolism and cytoplasmic vacuolization, a pattern frequently reported in environmental xenobiotic exposure [17, 21–23].

### Inflammatory pathways and hepatic injury

The activation of inflammatory signaling cascades, especially the NF-κB pathway, has been widely implicated in chemical-induced hepatotoxicity. Upregulation of pro-inflammatory cytokines such as TNF-α and interleukin-6 intensifies hepatic injury by recruiting immune cells and amplifying necrotic progression [4, 24]. During this process, the initial oxidative stress induced by styrene exposure triggers lipid peroxidation and mitochondrial dysfunction, leading to the release of damage-associated molecular patterns, which further activate Kupffer cells and endothelial cells. These activated cells produce additional cytokines and reactive oxygen species, sustaining a feed-forward inflammatory loop that aggravates hepatocellular degeneration. Prolonged activation of these pathways compromises membrane integrity, disrupts hepatic microcirculation, and promotes sinusoidal and perivascular lesions, ultimately resulting in hepatocyte necrosis and fibrosis. This inflammatory response is crucial in determining whether hepatic changes are adaptive or pathological. Persistent cytokine activation appears to drive hepatocellular degeneration, consistent with findings in *D. rerio* and *Gobiocypris rarus* exposed to styrene and polystyrene microplastics [4, 17].

### Metabolic activation of styrene and oxidative stress mechanisms

Styrene metabolism in fish is primarily mediated by cytochrome P450 enzymes, particularly via the AhR–CYP1A1 axis, which facilitates detoxification while simultaneously generating electrophilic intermediates, such as styrene oxide, that intensify oxidative stress and hepatocellular injury [17]. Comparative analyses in *O. latipes* and *D. rerio* confirm that AhR–CYP1A1 activation and subsequent oxidative stress responses occur at comparable concentration ranges, underscoring interspecies conservation of styrene’s toxicodynamics [4, 17, 19, 25]. Our data further indicate that transcriptional activation of CYP1A1 and antioxidant genes preceded the onset of structural lesions, such as vacuolization and necrosis, suggesting that these molecular endpoints are early biomarkers of sublethal hepatotoxicity.

### Antioxidant response and limits of cellular protection

Similar dual regulatory patterns have been reported in *O. latipes* and *D. rerio*, where elevated CYP1A1, SOD, and TNF expression coincided with histopathological lesions, supporting the cross-species relevance of these molecular markers [1, 21, 26]. Nevertheless, the progressive increase in oxidative markers at higher styrene concentrations implies that antioxidant defense systems became insufficient to counteract excessive ROS production, leading to oxidative imbalance and hepatocyte degeneration [4, 22, 27, 28].

### Integrated mechanistic pathways of styrene hepatotoxicity

Our findings confirm that styrene-induced hepatotoxicity is a multifactorial process involving oxidative stress, immune activation, and metabolic imbalance. The pronounced upregulation of CYP1A1 observed in *O. celebensis* reflects the activation of the AhR signaling cascade [19, 29], which is consistent with previous studies [17, 30, 31] reporting AhR-mediated phase I detoxification in aromatic hydrocarbon exposure. Following ligand binding, the AhR–CYP1A1 complex translocates to the nucleus, inducing CYP1A1 transcription and increasing ROS production through styrene oxidation. The elevated catalase and SOD expression indicates the activation of antioxidant defense systems that counteract excessive ROS, yet persistent oxidative stress sustains inflammatory signaling. This was further evidenced by increased TNF expression, highlighting the role of NF-κB–mediated inflammation in amplifying hepatic damage [31–33].

### Ecophysiological and population-level implications

Chronic hepatic impairment may compromise fish growth, energy allocation, and reproductive success, ultimately affecting population dynamics and ecosystem health in polluted habitats [17, 22]. Styrene exposure triggers a sequential cascade at the mechanistic level, beginning with ROS formation and lipid peroxidation, followed by cytokine release and Kupffer cell activation. Collectively, these responses impair hepatic microcirculation, disrupt endothelial integrity, and promote necrosis. Such molecular and cellular events align with previous reports describing styrene-induced peroxidative and inflammatory injury in zebrafish and rare minnow models [4, 6, 19, 34].

### Correlation between biomarkers and histological damage

The strong correlations between gene expression profiles and histopathological alterations further validate the role of molecular biomarkers as early indicators of hepatic stress. CYP1A1 showed the highest r_2_, reflecting its role as a sensitive marker for xenobiotic metabolism and hepatocellular burden. The combined activation of AhR-CYP1A1 and Nrf2-ARE pathways illustrates a dual process in which detoxification and oxidative stress coexist, ultimately aggravating liver injury [6, 17, 19]. Similarly, TNF upregulation indicates that inflammation directly contributes to hepatocellular necrosis, confirming a link between molecular signaling and tissue pathology [5, 33, 34]. CYP1A1 activation through the AhR pathway initiates styrene metabolism, yielding reactive intermediates that promote oxidative stress. The Nrf2–ARE axis is upregulated as a compensatory response, enhancing antioxidant defenses (e.g., catalase, SOD). Persistent ROS and intermediate overload activate NF-κB signaling, driving TNF-α expression and inflammation [35, 36]. The integration of these pathways underlines the co-operation of detoxification, oxidative stress, and immune activation in hepatotoxicity (Figure 3).

### Relevance of *O. celebensis* as a tropical sentinel species

The use of *O. celebensis* provides a comparative advantage for tropical ecotoxicology, as its native physiology and sensitivity to xenobiotic exposure reflect realistic environmental conditions in Southeast Asian freshwater habitats. The integration of molecular biomarkers, such as CYP1A1, catalase, SOD, and TNF, into monitoring programs could enhance the early detection of pollutant stress and inform ecological risk assessment strategies in tropical aquatic systems. Collectively, these results emphasize that the liver of *O. celebensis* serves as a responsive and ecologically relevant model for assessing styrene-induced toxicity. The integration of molecular and histopathological endpoints establishes a mechanistic framework for evaluating PDPs in tropical aquatic ecosystems [22, 23]

### Broader ecological and One Health implications

Beyond these mechanistic insights, this study’s findings also hold broader ecological and One Health significance. The hepatotoxic responses observed in *O. celebensis* illustrate how styrene contamination in aquatic environments transcends ecological boundaries, posing interconnected risks to animal and human health. Styrene released from industrial effluents can bioaccumulate through the aquatic food web, leading to genotoxic, endocrine, and immunotoxic disturbances across trophic levels and ultimately threatening food safety and public health through fish consumption [37, 38]. In Southeast Asia, where industrial regulation and waste management remain inconsistent, styrene concentrations in surface waters often exceed international safety thresholds established by the WHO and the USEPA [34, 36]. These findings underscore the urgent need for integrated surveillance and policy interventions that link environmental monitoring with health protection. This study provides a mechanistic foundation for early detection of xenobiotic stress by employing molecular biomarkers such as CYP1A1, SOD, and TNF, thereby advancing the implementation of One Health-based risk assessment and ecosystem management strategies in tropical regions [25, 39].

## CONCLUSION

This study demonstrated that acute exposure to environmentally relevant concentrations of styrene induces marked hepatotoxicity in *O. celebensis* through coordinated histopathological and molecular disruptions. Concentration-dependent hepatic alterations, including cytoplasmic vacuolization, congestion, inflammatory infiltration, and necrosis, were most pronounced at 0.5 and 0.75 mg/L, indicating substantial cellular degeneration. These tissue-level injuries closely correlated with strong upregulation of key biomarker genes, particularly CYP1A1 (up to 36.9-fold), catalase, SOD, and TNF, reflecting activation of the AhR–CYP1A1 detoxification pathway, Nrf2-mediated antioxidant defenses, and NF-κB–driven inflammatory responses. The strong correlations between lesion severity and biomarker activation further confirm the mechanistic linkage between styrene metabolism, oxidative stress, inflammation, and hepatocellular injury.

Practical implications of these findings highlight the sensitivity of *O. celebensis* to plastic-derived monomers, supporting its suitability as a tropical sentinel species for freshwater ecotoxicological monitoring. Given that styrene levels in Southeast Asian rivers often exceed international safety thresholds, the biomarker suite identified here, CYP1A1, catalase, SOD, and TNF, offers a powerful early-warning toolbox for environmental risk assessment. These results underscore the urgent need for strengthened industrial waste management, regulatory surveillance, and One Health-oriented monitoring strategies in tropical regions where pollutant degradation kinetics differ significantly from temperate systems.

A key strength of this study lies in its integrated approach, combining histopathological scoring with molecular biomarker profiling under controlled exposure conditions, thereby providing mechanistic clarity and enhancing ecological relevance. The use of a native tropical species adds further value for region-specific risk assessment.

However, several limitations must be acknowledged. The study evaluated only short-term exposure and a single pollutant, whereas natural environments involve chronic, multi-contaminant scenarios. In addition, bioaccumulation dynamics, reproductive impacts, and long-term physiological consequences were not assessed. These constraints limit the ability to extrapolate fully to ecosystem-level outcomes.

Future research should therefore incorporate chronic and multigenerational designs, mixture toxicity assessments with microplastics and co-contaminants, trophic transfer experiments, and *in situ* field validations. Investigations into endocrine, reproductive, and behavioral endpoints would further refine the species’ utility as a comprehensive bioindicator.

The integration of molecular and histopathological evidence presented here confirms that styrene poses a significant hepatotoxic threat to tropical freshwater fish and validates *O. celebensis* as an effective sentinel organism for pollutant monitoring. These insights provide a stronger mechanistic foundation for environmental protection strategies and reinforce the One Health imperative to safeguard aquatic ecosystems, wildlife, and human health.

## DATA AVAILABILITY

The supplementary data can be made available from the corresponding author upon request.

## AUTHORS’ CONTRIBUTIONS

ARA: Conducted the experiments and wrote the initial draft. HSD and WM: Supervised the methodology and analysis. KY: Supervised the methodology and field sampling. MAN: Provided technical support. MRC: Contributed to data curation and manuscript revisions. All authors have read and approved the final version of the manuscript.
